# Evaluation of a transdiagnostic mental health intervention in German primary care: a parallel-group, two-arm, cluster randomised controlled pilot study

**DOI:** 10.1186/s12875-026-03377-4

**Published:** 2026-05-21

**Authors:** Christopher Ebert, Marie Vogel, Jochen Gensichen, Hanna Reif, Lukas Junker, Lena Grögor, Frank Padberg, Peter Falkai, Thomas Ehring, Alkomiet Hasan, Stefan Leucht, Kirsten Lochbühler

**Affiliations:** 1 Institute of General Practice and Family Medicine, University Hospital, LMU Munich, Nußbaumstraße 5, Munich, 80336 Germany; 2 DZPG (German Center for Mental Health), partner site Munich/Augsburg, Germany; 3 Department of Psychology, LMU Munich, Munich, Germany; 4 Department of Clinical Psychology and Psychotherapy, Psychologische Hochschule Berlin, Berlin, Germany; 5 Department of Psychiatry and Psychotherapy, University Hospital, LMU Munich, Munich, Germany; 6 Max Planck Institute of Psychiatry, Munich, Germany; 7 Department of Psychiatry, Psychosomatics and Psychotherapy, Medical Faculty, University of Augsburg, Augsburg, Germany; 8 Department of Psychiatry and Psychotherapy, TUM School of Medicine and Health, Technical University of Munich, Munich, Germany

**Keywords:** Primary Care, Family Medicine, General Practitioner, Transdiagnostic, Unified Protocol, Brief Psychological Intervention, Psychoeducation, Mental Health, Pilot Study, Randomised Controlled Trial

## Abstract

**Introduction:**

Adequate mental health treatment in primary care (PC) is often hindered by structural factors, such as time constraints, and the high comorbidity of mental health conditions. A transdiagnostic approach may help address these challenges, yet evidence on interventions delivered by general practitioners (GPs) remains limited. This pilot study therefore assessed the feasibility, acceptability and potential effectiveness of a GP-led transdiagnostic intervention.

**Methods:**

PC practices in Munich (Germany) were randomised (1:1) to an intervention group (IG) or a control group (CG). In the IG, GPs delivered the transdiagnostic intervention focused on psychoeducation, cognitive flexibility and emotion-based avoidance. CG GPs provided improved treatment as usual based on diagnosis-specific clinical guidelines. In both groups, treatment comprised four 20-min sessions. Feasibility and acceptability (primary outcome) were assessed post-treatment from patients and GPs across key treatment aspects: recruitment, delivery, response, maintenance and unintended consequences. Potential effectiveness (secondary outcome) was observed through patients’ pre-post changes in transdiagnostic factors – beliefs about emotions, cognitive reappraisal, emotion suppression, experiential avoidance and negative affectivity – as early indicators of symptom change.

**Results:**

21 PC practices participated (IG: 11; CG: 10), recruiting 87 patients (mean age: 47 years; 68% female), with 77 (IG: 46; CG: 31) completing treatment. Feasibility and acceptability ratings tended to favour the transdiagnostic intervention. More IG GPs stated a better treatment integration into daily care, greater utility to bridge time until psychotherapy and higher satisfaction with the structure, content as well as perceived patient benefit. Similarly, IG patients rated the treatment structure and content clarity more positively. Relative to CG patients, a trend of greater improvements in transdiagnostic factors over time was observed in IG patients – most pronounced in beliefs about emotions (Emotion Beliefs Questionnaire: -8.89, 95% CI [-15.10, -2.74]), cognitive reappraisal (Emotion Regulation Questionnaire subscale: 6.03, 95% CI [2.52, 9.55]) and experiential avoidance (Brief Experiential Avoidance Questionnaire: -4.57, 95% CI [-8.69, 0.09]).

**Discussion:**

The findings support the progression to a fully powered main trial. Future research should optimise recruitment strategies, streamline the intervention and improve outcome monitoring.

**Trial registration:**

The study has been registered with the German Clinical Trials Register: 18^th^ of March 2024, https://drks.de/search/en/trial/DRKS00033386.

**Supplementary Information:**

The online version contains supplementary material available at 10.1186/s12875-026-03377-4.

## Background

Mental health conditions impose a major burden on society [1], affecting 13% of the global population in 2019 – most commonly through anxiety and depressive disorders [2]. However, only a fraction of affected individuals ever receive psychotherapy [3], as demand exceeds capacity [4]. Since early treatment is associated with improved outcomes [5], primary care (PC) plays an important role in bridging the existent gap between symptom onset and the initiation of psychotherapy [6].

Addressing mental health conditions in PC is constrained by structural factors such as limited consultation time [7] and volume-based reimbursement [8], while the high comorbidity of mental health conditions further complicates treatment [9].

Currently, pharmacological treatment remains the predominant approach used by general practitioners (GPs) [4, 10], despite evidence that mild to moderate mental health symptoms – most prevalent in PC [11] – may respond better to brief psychotherapeutic interventions [12], which also align more closely with patient preferences [13].

To better integrate psychological interventions into PC, a transdiagnostic approach has been deemed promising [14]. In contrast to disorder-specific classification systems – considered misaligned with routine GP practice [10, 15] – transdiagnostic treatments can be applied across diverse symptom presentations [4, 16], particularly in early stages [17]. By targeting shared underlying mechanisms (i.e., transdiagnostic factors), a transdiagnostic approach is proposed to address comorbidity more appropriately [16]. Furthermore, by streamlining therapist training [18] and accommodating brief session formats [12], it may be particularly suitable to help close the mental health treatment gap [19].

Beyond their inherent practical advantages, transdiagnostic approaches have been shown to be superior to usual care and waitlist control conditions and at least as effective as disorder-specific treatments in specialised settings [19, 20]. In PC, a recent meta-analysis demonstrated the efficacy of transdiagnostic interventions [21], with selected large-scale trials providing particularly relevant examples of their potential. For instance, the PsicAP study evaluated a seven-session group-based transdiagnostic CBT intervention in Spanish PC patients (*n* = 1061) and demonstrated superiority over usual care [22]. Similarly, Newby et al. [23] examined a six-session internet-delivered transdiagnostic intervention in Australian PC patients (*n* = 2109), showing comparable or greater efficacy relative to disorder-specific treatments. Notably, group-based therapy may be difficult to integrate into routine PC [24], while internet-delivered approaches may not fully utilise the therapeutic potential of the GP-patient relationship [10]. When specifically considering face-to-face transdiagnostic interventions delivered directly by GPs, only four of the 38 studies included in the above-mentioned meta-analysis were identified [25–28], with substantial heterogeneity in treatment procedures and outcomes. To address this, we developed a brief GP-delivered transdiagnostic intervention for psychological first aid in PC [29], adapted from the Unified Protocol for Transdiagnostic Treatment of Emotional Disorders (UP) [30].

The UP – a CBT-based manual – has demonstrated efficacy across various settings and mental health conditions [31, 32] and has been endorsed for implementation in PC [14]. It targets core transdiagnostic mechanisms, such as psychoeducation, mindfulness, cognitive flexibility, interoceptive exposure/anxiety sensitivity and countering emotion-based avoidance/situational exposure, in order to improve emotion regulation [33]. These mechanisms are considered central across a wide range of mental health conditions [34–38] and are thought to function as mutually reinforcing processes underlying psychopathology: (1) frequent experiences of negative affect may foster (2) maladaptive emotional beliefs (e.g., perceiving emotions as aversive or uncontrollable), which in turn promote (3) dysfunctional cognitive and behavioural coping strategies such as rumination and experiential avoidance [39]. Targeting these mechanisms is therefore particularly relevant for reducing maladaptive responses to negative emotions [40] and, ultimately, the burden of internalising disorders [41] commonly managed in PC, including depressive, anxiety and somatoform symptoms [9].

Positive outcomes have been reported even when UP components are administered in a modularised form [42]. In line with PC-specific recommendations for psychotherapeutic treatments (4–6 sessions of ≤ 30 min [43]), the developed intervention comprises three core modules – psychoeducation, cognitive flexibility and countering emotion-based avoidance – plus a summary session; delivered across four 20-min appointments. Module selection was informed by established psychotherapeutic concepts in PC [40, 44–46] and by their feasibility for realisation in PC.

To date, the UP has only been implemented in PC in a group format, showing promising results [47, 48]. In response to Drury et al.’s [49] call for research on different delivery modes of the UP in PC, this cluster randomised controlled pilot study aimed to: (1) assess the acceptability and feasibility of a GP-led transdiagnostic intervention based on patient and GP experiences with treatment recruitment, delivery, response, maintenance and unintended consequences; (2) explore its potential effectiveness through changes in transdiagnostic factors (i.e., beliefs about emotions, cognitive reappraisal, emotion suppression, experiential avoidance and negative affectivity) as early indicators of symptom change.

## Methods/design

### Study design and participants

The pilot study followed a parallel-group, two-arm, cluster randomised controlled design. Participants were randomly allocated (1:1 ratio) to one of two treatment groups, either the transdiagnostic intervention (i.e., intervention group, IG) or improved treatment as usual (iTAU; i.e., control group, CG). Data collection took place at baseline (t_0_: patients only), after individual treatment completion (t_1_: patients) and after all treatments were completed (t_2_: GPs).

Inclusion criteria for GPs were (1) a qualification in psychosomatic PC^1^ or an additional psychiatric training and (2) written informed consent. Participants had to (1) be fluent in German, (2) be at least 18 years old, (3) demonstrate psychological distress and (4) provide written/digital informed consent. GPs excluded patients if any of the following applied: (1) life expectancy ≤ 12 months, (2) current substance abuse/dependency (e.g., alcohol, illicit drugs or medication; excluding nicotine dependency), (3) risk of suicidality, (4) cognitive impairment (e.g., dementia), (5) severe mental disorder (e.g., severe depression, bipolar disorder, borderline disorder), (6) already receiving psychotherapy at study start, (7) change in psychotropic medication intake within 6 weeks before study start or (8) physical impairments preventing them from visiting the PC practice for in-person treatment.

The pilot study was approved by the ethics committee at LMU Munich (01^st^ of March 2024; ref no. 24–0080) and was registered in the German Clinical Trials Register (Deutsches Register Klinischer Studien [DRKS]; trial registration number DRKS00033386; 18^th^ of March 2024). It complied with the Helsinki Declaration [50] and followed the Consolidated Standards of Reporting Trials (CONSORT) extension to randomised pilot and feasibility studies [51] as well as the Standard Protocol Items: Recommendations for Interventional Trials (SPIRIT) [52]. Prior to completion of participant recruitment, a study protocol was published [29].

### Recruitment, randomisation and blinding

From February to October 2024, GPs from PC practices in or around Munich (Germany) were recruited via telephone, mail and email using the teaching practice network of the Institute of General Practice at the LMU Munich university hospital. For eligible PC practices, cluster randomisation was performed by a study-independent member of the study team using a computer-generated sequence in a 1:1 ratio. Randomisation was stratified by the number of participating GPs per practice to account for the likelihood that practices with more GPs would recruit a higher number of patients. Between June 2024 and April 2025 new and ongoing patients were recruited by GPs based on their clinical judgement, supplemented by a brief psychological distress questionnaire (i.e., Kessler-6 (K-6) [53]) with age-specific German norm values [54]. This approach was chosen to reflect routine PC practice, where patient identification commonly relies on a GP’s intuitive expertise [15], while additionally providing a standardised patient selection framework. Given the intervention’s intended role as a psychological first-aid approach, broad psychological distress rather than disorder-specific criteria was used to identify patients. Eligibility was assessed by GPs using a checklist. Further details on recruitment and randomisation were provided in the study protocol [29]. Blinding of researchers, GPs and patients was not feasible due to the study design; however, patients were not informed about which treatment was considered the active intervention.

### Procedure

**Fig. 1:**
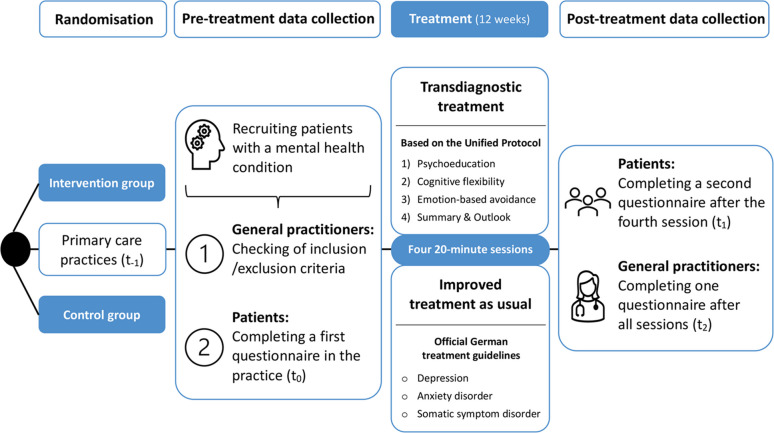
Study design and procedures

A two-hour training was given to practices of both study groups before treatment initiation (t_−1_), informing GPs about the study procedure (identical for IG and CG) and treatment content (IG: transdiagnostic treatment; CG: iTAU). At enrolment (t_0_: baseline), patients in both groups completed a first questionnaire digitally or on paper in their PC practice. Treatment lasted up to 12 weeks, during which patients received four 20-min sessions from their GP. In the IG, treatment followed the UP [30], using a table-flipchart to sequentially deliver adapted versions of the UP modules “understanding emotions” (module 2), “cognitive flexibility” (module 4) and “countering emotion-based avoidance” (module 5), concluded by a summary session. In addition, patients were assigned session‑related homework, including exercises and reading material from a supplementary handbook. CG patients received iTAU, in which GPs were instructed to deliver their usual care while explicitly orienting treatment on provided extracts from official German diagnosis-specific clinical guidelines for depression, anxiety and somatic symptom disorders [55–57]. This approach was chosen to reduce heterogeneity inherent in unrestricted TAU while preserving routine GP practice through a minimally standardised guideline-informed framework. A detailed account on treatment content can be found in the study protocol [29]. After the fourth treatment session (t_1_), participants completed a second questionnaire. GPs submitted a digital questionnaire after having treated all patients (t_2_) (Fig. 1). Patients were compensated with two €25 vouchers after completing each questionnaire, while GPs received €100 per recruited patient.

### Outcomes

#### Primary outcome measures

Feasibility and acceptability were assessed at t_1_ (patients) and t_2_ (GPs) following an established process evaluation framework for cluster randomised controlled trials [58] and items adapted from a previous pilot study [59]. Participants rated their agreement on statements related to different treatment aspects – recruitment, delivery, response, maintenance and unintended consequences – using a 4-point Likert scale (1: ‘strongly disagree’ to 4: ‘strongly agree’). In addition, they evaluated treatment total time effort (GPs only), treatment duration and session quantity (3-point Likert scales – 1: ‘too short/few/low’ to 3: ‘too long/many/high’) and provided an overall treatment satisfaction rating (6-point Likert scale – 1: ‘very good’ to 6: ‘very bad’). A full list of feasibility and acceptability items is outlined in additional file 1.

#### Secondary outcome measures

Secondary outcomes were obtained via patient self-report at t_0_ and t_1_. As interim measures of potential effectiveness, patients completed the following transdiagnostic questionnaires, each assessing specific components of the intervention: the Emotion Beliefs Questionnaire (EBQ; [60]) which assesses maladaptive beliefs about the controllability and usefulness of emotions using 16 items rated on a 7-point Likert scale (1 = strongly disagree to 7 = strongly agree), with a higher total score indicating more negative emotional beliefs (corresponding to session 1; Cronbach’s α = 0.87); the Emotion Regulation Questionnaire (ERQ; [61]), a 10-item measure rated on a 7-point Likert scale (1 = strongly disagree to 7 = strongly agree), comprising the subscales cognitive reappraisal and expressive suppression, with a higher score implying greater use of the respective strategy (cognitive reappraisal subscale corresponding to session 2; Cronbach’s α of cognitive reappraisal subscale = 0.84, Cronbach’s α of emotion suppression subscale = 0.73); the Brief Experiential Avoidance Questionnaire (BEAQ; [62]), which captures the tendency to avoid unwanted internal and external experiences using 15 items rated on a 6-point Likert scale (1 = strongly disagree to 6 = strongly agree), with a higher score reflecting greater experiential avoidance (corresponding to session 3; Cronbach’s α = 0.92); and the negative affectivity subscale of the Modified Personality Inventory for DSM-5 Brief Form (PID5BF + M; [63]), which assesses negative affectivity using six items rated on a 4-point Likert scale (0 = very false or often false to 3 = very true or often true), with a higher score indicating greater negative affectivity (reflecting the overarching transdiagnostic concept of the UP; Cronbach’s α = 0.74).

Further diagnosis-specific outcomes included the Patient Health Questionnaire-9 (PHQ-9; [64]), a 9-item measure of depressive symptoms over the past two weeks rated on a 4-point Likert scale (0 = not at all present to 3 = present nearly every day), with a higher score indicating greater symptom severity (Cronbach’s α = 0.95); the Generalised Anxiety Disorder Screener-7 (GAD-7; [65]), evaluating general anxiety symptoms over the past two weeks with seven items on a 4-point Likert scale (0 = not at all present to 3 = present nearly every day), where a higher score reflects greater anxiety severity (Cronbach’s α = 0.87); the Patient Health Questionnaire-15 (PHQ-15; [66]), capturing somatic symptom severity over the past four weeks via 15 items rated on a 3-point Likert scale (0 = not bothered at all to 2 = bothered a lot), with a higher score corresponding to greater somatic burden (Cronbach’s α = 0.60); and the Primary Care PTSD Screen for DSM-5 (PC-PTSD-5; [67]), a 5-item dichotomous (yes/no) screening tool for post-traumatic stress symptoms, with a higher score denoting a greater likelihood of PTSD (Cronbach’s α = 0.75; assessed only at t_1_).

#### Additional outcome measures

Socio-demographic data was collected from patients at t_0_ (i.e., age, sex, nationality, marital status, education, employment, psychiatric history) and from GPs at t_2_ (i.e., age, sex), along with occupation-related data (i.e., professional experience, additional qualifications, practice characteristics). At t_1_, patients self-reported medication prescribed during treatment. In addition, those with a chronic illness completed the Patient Assessment of Chronic Illness Care (PACIC) short form [68], an 11-item questionnaire assessing the quality of care received for chronic conditions over the past six months, rated on a 4-point percentage scale (0–25% to 76–100%). Throughout the study, GPs recorded the total number of patients approached, treatment fidelity, serious adverse events and reasons for patient withdrawal.

### Statistical methods

#### Sample size

Considering time and staff resources, the pilot study aimed to recruit 20 PC practices, each enrolling four to six patients, targeting a total sample of 100 patients, with an expected drop-out rate of 20%. This follows Eldridge et al. [69], who suggested that for pilot cluster randomised trials – assuming an intra-class correlation (ICC) of 0.05 [70] – either a fixed cluster number of 20 with six participants each or a fixed cluster size of five participants with a total of 23 clusters are required to keep the maximum potential error in any estimated rate – e.g., relating to treatment recruitment, retention, response – below 10%. In line with previous research [71], the recruitment target of six patients per practice was chosen to consider the realistic recruitment capacity of PC practices within a study context.

#### Statistical analysis

The software R [72] was used for data analyses, including the packages “psych” [73], “lavaan” [74], “marginaleffects” [75] and “lme4” [76].

Reliability of questionnaires was assessed using Cronbach’s alpha (α). Descriptive statistics summarised sample characteristics. Feasibility and acceptability outcomes were visualised graphically for both study groups. Linear mixed models (LMM) with restricted maximum likelihood estimation (REML) were applied to calculate within- and between-group changes over time in both transdiagnostic outcomes (EBQ, ERQ, BEAQ, PID‑5‑BF + M) and disorder‑specific outcomes (PHQ‑9, GAD‑7, PHQ‑15). Treatment group (IG vs. CG), time (pre vs. post) and their interaction were specified as fixed effects. To account for clustering within PC practices, a random effect for patients’ practice affiliation was included. A random slope for the interaction between practice and time was initially considered but was omitted due to convergence issues related to the small sample size and minimal between-practice variance. Age, gender and educational level were included in all LMM as covariates. In line with the study's exploratory nature and limited statistical power, treatment effects over time were reported with 95% confidence intervals (CIs) obtained via parametric bootstrap (5000 iterations); no p‑values were interpreted [51, 77].

In addition, rates of caseness, recovery, reliable improvement, reliable deterioration and reliable recovery were calculated following Cano-Vindel et al. [22]. Caseness was defined as meeting the baseline clinical threshold on at least one measure of depressive, anxiety or somatoform symptoms: PHQ-9 ≥ 10; GAD-7 ≥ 10; PHQ-15 ≥ 3 of the first 13 items scored as “bothered a lot”. Recovery required a transition from baseline caseness to below the clinical threshold on all measures post-treatment. Given the pilot design and limited power, reliable improvement and deterioration could not be calculated using study-specific indices but were instead based on the established scale-specific thresholds reported by Cano-Vindel et al. [22] (PHQ-9: ± 6; GAD-7: ± 5; PHQ-15: ± 6). Reliable improvement required threshold-specific symptom improvement without simultaneous reliable deterioration on another measure, and vice versa. Reliable recovery was determined as recovery plus reliable improvement on at least one measure.

The analyses included all participants with baseline and post treatment data (*n* = 77; IG: 46, CG: 31). Due to the minimal amount of missing outcome data (1.16%), no imputation was performed.

## Results

### Socio-demographics

#### GPs

The mean age of GPs was 48.3 years (*SD* = 10.1) and 71% were female. Most (63%) had more than six years of clinical experience, with ≥ 15 years of experience being more common in IG GPs (54% vs. CG: 27%). Additional medical training (e.g., palliative care, sports medicine) was reported by 63% of GPs, but was more frequent in CG GPs (82% vs. IG: 46%). Further occupation-related data is shown in Table 1.

**Table 1:** Socio-demographic characteristics of GPs

Variable	Group		
	Intervention group (*n* = 13)	Control group (*n* = 11)	Total (*n* = 24)
	M/Count (*SD*/%)	M/Count (*SD*/%)	M/Count (*SD*/%)
Age, years	50.00 (*11.07*)	46.36 (*8.92*)	48.33 (*10.10*)
Gender			
Female	9 (69%)	8 (73%)	17 (71%)
Male	4 (31%)	3 (27%)	7 (29%)
Professional experience			
< 1 year	0	1 (9%)	1 (4%)
1—5 years	4 (31%)	4 (36%)	8 (33%)
6—10 years	2 (15%)	2 (18%)	4 (17%)
11—15 years	0	1 (9%)	1 (4%)
> 15 years	7 (54%)	3 (27%)	10 (42%)
Type of practice			
Individual practice	3 (23%)	5 (45%)	8 (33%)
Joint practice	1 (8%)	1 (9%)	2 (8%)
Group practice	6 (46%)	5 (45%)	11 (46%)
Medical care centre	3 (23%)	0	3 (13%)
Additional medical training (yes)	6 (46%)	9 (82%)	15 (63%)
Employed GPs per practice			
1	2 (15%)	1 (9%)	3 (13%)
2	1 (8%)	0	1 (4%)
3	6 (46%)	6 (55%)	12 (50%)
4	0	2 (18%)	2 (8%)
5	0	0	0
> 5	4 (31%)	2 (18%)	6 (25%)
Treated patients per quarter year			
≤ 500	0	0	0
≤ 1.000	2 (15%)	0	2 (8%)
≤ 1.500	4 (31%)	2 (18%)	6 (25%)
≤ 2.000	2 (15%)	6 (55%)	8 (33%)
> 2.000	5 (38%)	3 (27%)	8 (33%)

*n* = sample size, M = mean, *SD* = Standard Deviation

#### Patients

Patients’ mean age was 46.98 years (*SD* = 15.21), with IG patients being younger on average (43.38 years, *SD* = 13.66 vs. CG: 52.31 years, *SD* = 16.00). Most patients identified as female (68%). Regarding a previously diagnosed mental disorder, 33% reported depression, 8% anxiety disorder and 1% somatic symptom disorder; 32% had previously received mental health treatment. At baseline, average symptom scores were similar between groups for psychological distress (Kessler-6: IG = 13.40, CG = 13.29), anxiety (GAD-7: IG = 12.12, CG = 11.54) and somatoform symptoms (PHQ-15: IG = 12.77, CG = 12.80). CG patients scored slightly lower for depression symptoms (PHQ-9: IG = 14.44, CG = 12.83). Table 2 provides additional socio-demographic data.

**Table 2:** Socio-demographic characteristics of patients at baseline

Variable	Group		
	Intervention group (*n* = 52)	Control group (*n* = 35)	Total (*n* = 87)
	M/Count (*SD*/%)	M/Count (*SD*/%)	M/Count (*SD*/%)
Age, years	43.38 (*13.66*)	52.31 (*16.0*)	46.98 (*15.21*)
Patient Health Questionnaire			
PHQ-9	14.44 (*5.91*)	12.83 (*5.46*)	13.79 (*5.75*)
GAD-7	12.12 (*4.89*)	11.54 (*5.44*)	11.89 (*5.10*)
PHQ-15	12.77 (*5.31*)	12.80 (*5.73*)	12.78 (*5.45)*
Gender			
Female	36 (69%)	23 (66%)	59 (68%)
Male	16 (31%)	11 (31%)	27 (31%)
Missing	0	1 (3%)	1 (1%)
Nationality			
German	48 (92%)	31 (89%)	79 (91%)
Other	4 (8%)	4 (11%)	8 (9%)
Marital status			
Single	10 (19%)	11 (31%)	21 (24%)
Relationship/married	33 (63%)	15 (43%)	48 (55%)
Divorced	5 (10%)	6 (17%)	11 (13%)
Widowed	3 (6%)	2 (6%)	5 (6%)
Other	1 (2%)	0	1 (1%)
Missing	0	1 (3%)	1 (1%)
Level of education			
No school-leaving certificate	1 (2%)	0	1 (1%)
Lower secondary school diploma	8 (15%)	9 (26%)	17 (20%)
Secondary school diploma	13 (25%)	12 (34%)	25 (29%)
High school diploma	29 (56%)	14 (40%)	43 (49%)
Other	1 (2%)	0	1 (1%)
Vocational/professional training			
No vocational training	4 (8%)	6 (17%)	10 (11%)
Vocational training	23 (44%)	15 (43%)	38 (44%)
Vocational college/university degree	20 (38%)	9 (26%)	29 (33%)
Other	5 (10%)	5 (14%)	10 (11%)
Occupation			
Employed	45 (87%)	27 (77%)	72 (83%)
Not employed	7 (13%)	8 (23%)	15 (17%)
Previously diagnosed mental disorder			
Depression	16 (31%)	13 (37%)	29 (33%)
Anxiety disorder	3 (6%)	4 (11%)	7 (8%)
Somatic symptom disorder	0	1 (3%)	1 (1%)
Previous psychological treatment	14 (27%)	14 (40%)	28 (32%)

*n* sample size, *M* mean, *SD* Standard Deviation, *PHQ-9* Patient Health Questionnaire-9, *GAD-7* Generalized Anxiety Disorder-7, *PHQ-15* Patient Health Questionnaire-15; “not employed” included patients who were unemployed, retired, housewives/househusbands or studying

### Feasibility and acceptability

**Fig. 2:**
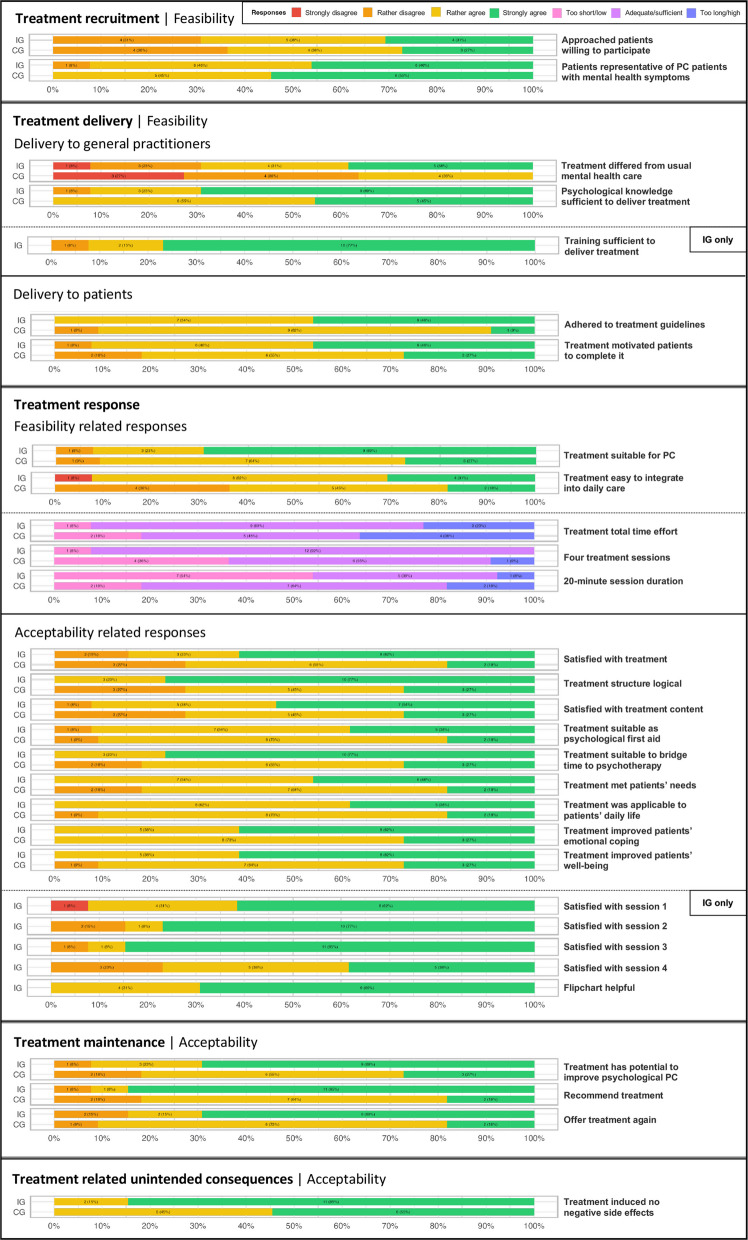
General practitioners’ ratings of treatment feasibility and acceptability. Legend: IG = Intervention group, CG = Control group, PC = Primary care, Percentages were rounded up (≥.5) or down to the nearest whole number

**Fig. 3:**
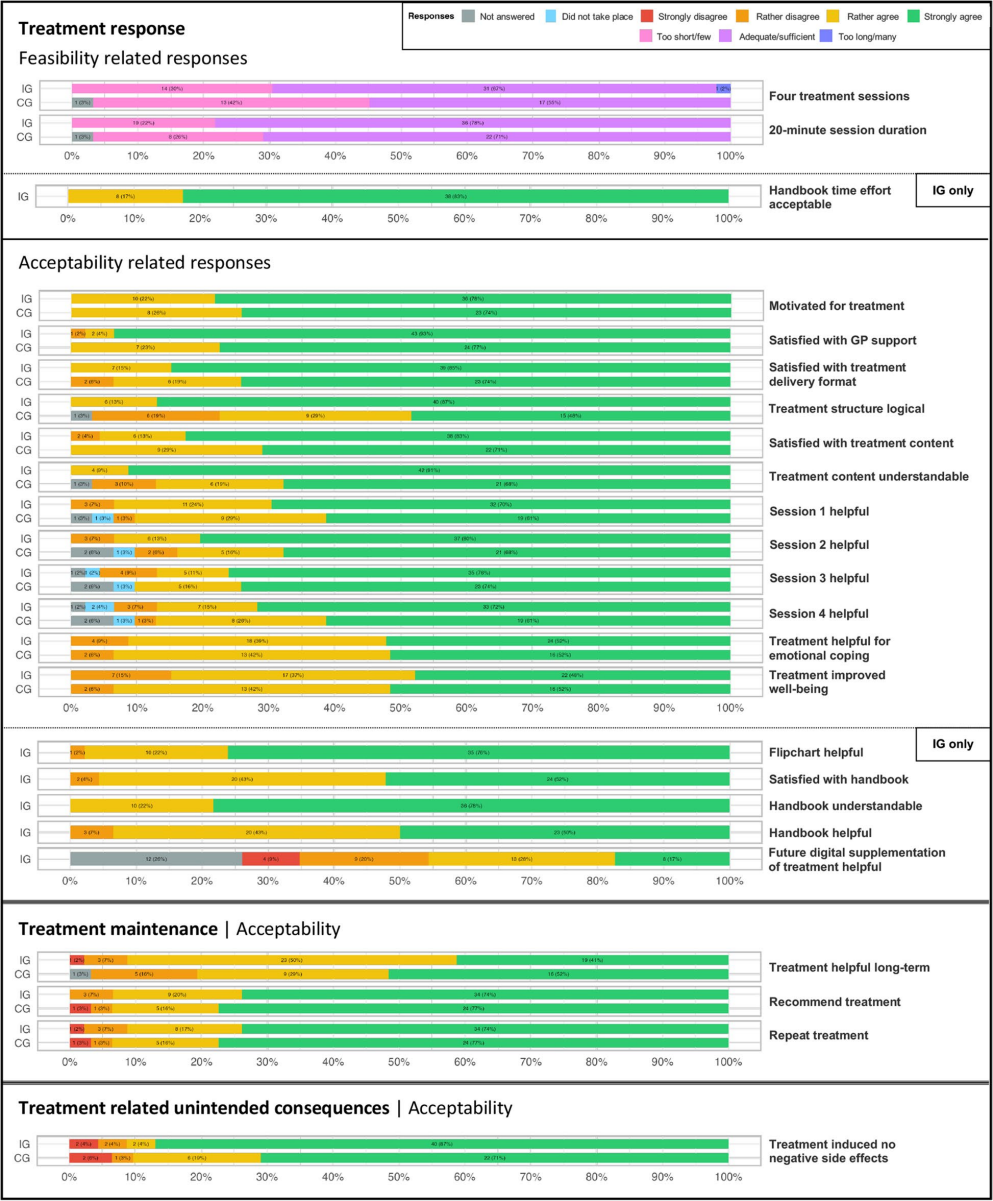
Patients’ ratings of treatment feasibility and acceptability. Legend: IG = Intervention group, CG = Control group, GP = General practitioner. Percentages were rounded up (≥.5) or down to the nearest whole number

Notable findings for feasibility and acceptability in terms of treatment recruitment, delivery, response, maintenance and unintended consequences are summarised below. Detailed ratings of GPs and patients for the transdiagnostic intervention and iTAU are presented in Figs. 2 and 3, respectively.

### Feasibility

#### Treatment recruitment

##### GP recruitment:

**Fig. 4:**
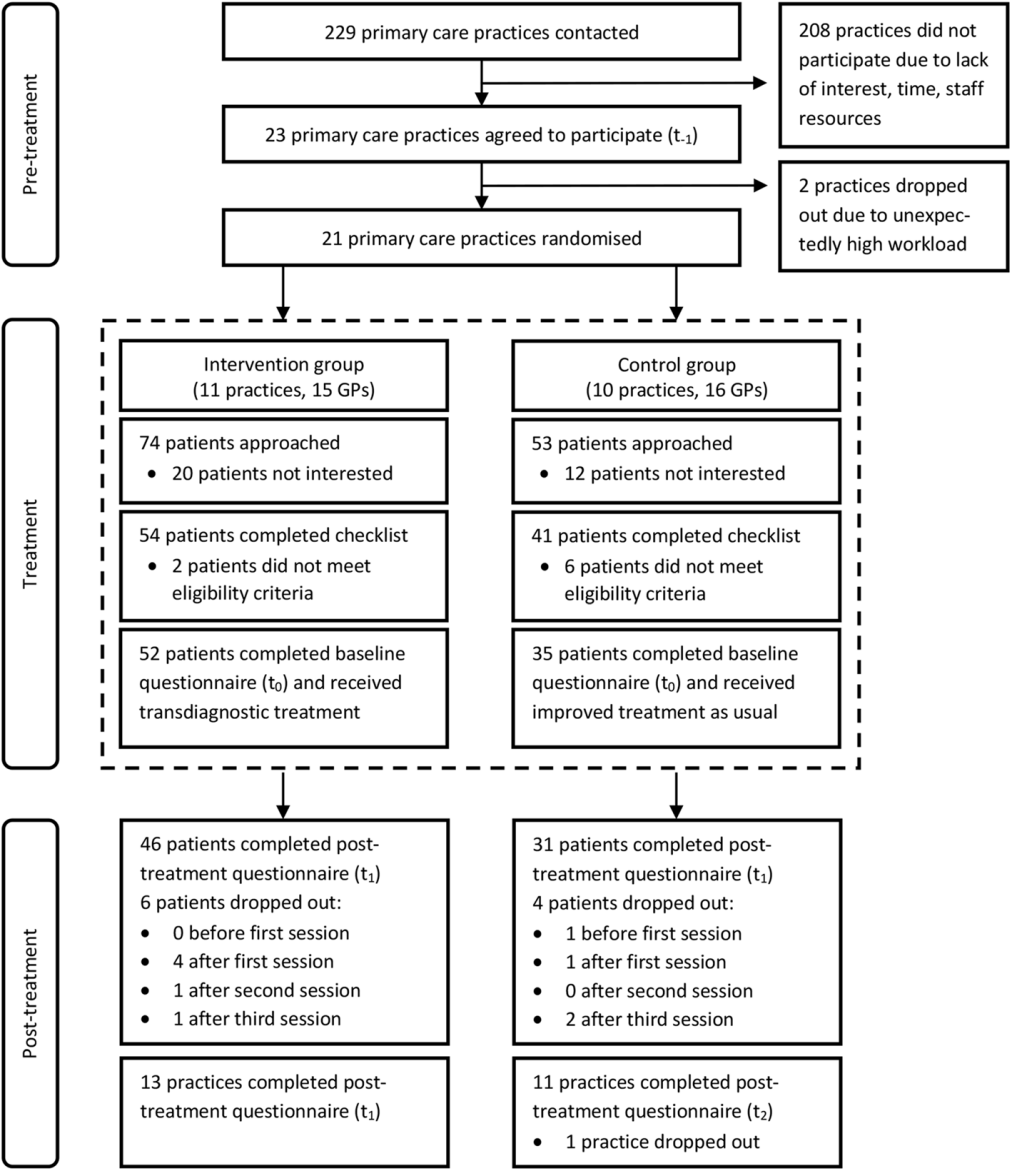
Flow chart of patients and general practitioners

Of 229 practices contacted, 23 initially agreed to participate. The most common reasons for non-participation were lack of interest, time constraints or insufficient staff capacity. Two practices withdrew prior to randomisation due to unexpectedly high workload. Eleven practices (15 GPs) were randomised to the IG and ten practices (16 GPs) to the CG. One CG practice discontinued participation during the study period (Fig. 4).

##### Patient recruitment:

127 patients with mental health symptoms were approached by GPs, 95 expressed interest and 87 met the eligibility criteria (IG: 52, CG: 35). Of these, 77 completed both the pre- and post-treatment assessments (IG: 46, CG: 31), while ten dropped out (IG: 6, CG: 4) without providing specific reasons for terminating their participation. One IG practice and three CG practices did not recruit any patients, citing increased workload, structural changes, staff shortages or other external obligations (Fig. 4).

The majority of GPs in both groups (IG: 69%, CG: 63%) agreed^2^ that approached patients’ willingness to participate was high. In related open-ended responses, GPs identified existing trust and presenting the intervention as a source of relief or a bridge to psychotherapy as key recruitment facilitators. Suggested improvements included raising public awareness of mental health treatment, displaying posters in waiting rooms and offering video consultations.

Apart from one IG GP, all GPs (IG: 92%, CG: 100%) reported that recruited patients were representative of typical PC patients with mental health symptoms.

#### Treatment delivery

##### Delivery to GPs:

A higher proportion of IG GPs (69% vs. CG: 36%) indicated that the treatment differed from their usual mental health care. Based on the available data from corresponding open-ended responses, IG GPs described their usual care as involving diagnostic tools, supportive conversations and referrals to specialists or psychotherapists, sometimes supplemented by pharmacological treatment. CG GPs similarly reported personalised approaches, comprising attentive listening, supportive conversations and pharmacological prescriptions. With the exception of one IG GP, all GPs (IG: 92%, CG: 100%) felt that their psychological knowledge was sufficient to administer the treatment. In the IG, most GPs (92%) perceived the 2‑hour training as sufficient preparation for treatment delivery.

##### Delivery to patients:

All GPs but one CG GP (IG: 100%, CG: 91%) reported adherence to treatment guidelines. In additional open‑ended responses, seven IG GPs reported going beyond the protocol, mostly by engaging in extended discussions about patients’ current situations. In the CG, three CG GPs reported supplementing official guidelines with their own psychotherapeutic experience and adopting a non-directive (patient-centred) approach in consultations. In addition, information on concurrent pharmacological treatment was available for 41 IG patients and 17 CG patients (missing data: IG = 4; CG = 14). Based on the available data, psychotropic medication prescription was reported by nine IG patients and three CG patients. In the IG, documented medications included herbal treatment (St John’s wort), SSRIs (escitalopram, sertraline, citalopram), SNRIs (venlafaxine, duloxetine), anxiolytic medication (opipramol), stimulant medication (methylphenidate), sedative-hypnotic medication (clomethiazole), while the CG listed herbal treatment (St John’s wort), SSRIs (sertraline) and NaSSAs (mirtazapine). Clearly therapeutic-standard dosing was documented for one patient in each group (IG patient: methylphenidate 50 mg, venlafaxine 150 mg; CG patient: mirtazapine 30 mg).

A higher share of IG GPs (92% vs. CG: 82%) agreed that the treatment motivated patients to complete it. Across the treatment period, 88% of IG patients completed all four sessions (session 1: 100%, session 2: 92%, session 3: 90%), compared to 89% in the CG (session 1: 97%, session 2: 94%, session 3: 94%) (Fig. 4).

#### Treatment response

##### Feasibility-related GP responses:

Most GPs (IG: 92%, CG: 91%) considered the treatment suitable for PC, whereas more IG GPs (93% vs. CG: 63%) agreed that the treatment was easy to integrate into daily care. A greater proportion of IG GPs (69% vs. CG: 45%) rated the overall time effort for treatment as adequate. Nearly all IG GPs (92%) considered four sessions sufficient, compared to 55% of CG GPs; 36% of CG GPs regarded the number as too low. The 20-min session length was more frequently perceived as too short for content delivery by IG GPs (54% vs. CG: 18%), while most CG GPs (64% vs. IG: 38%) found it adequate.

##### Feasibility-related patient responses:

IG patients more often regarded the number (67% vs. CG: 55%) and length (78% vs. CG: 71%) of sessions as adequate. A substantial minority in both groups would have preferred more (IG: 30%, CG: 42%) and longer sessions (IG: 22%, CG: 26%).

In the IG, patients reported an average adherence of 94% to handbook‑related homework, with all agreeing that the associated time effort was acceptable.

### Acceptability

#### Treatment response

##### Acceptability-related GP responses:

More IG GPs (85% vs. CG: 73%) expressed satisfaction with the treatment overall and with the treatment content (92% vs. CG: 72%). All IG GPs deemed the treatment structure logical, relative to 72% of CG GPs. Among IG GPs, satisfaction with treatment sessions was generally high, with the highest level of agreement indicated for the first and third sessions (93% each), followed by the second (85%) and fourth sessions (76%). All IG GPs agreed that the flipchart was helpful for treatment delivery. In both groups, most GPs (IG: 92%, CG: 91%) agreed that the treatment was appropriate as psychological first aid. All IG GPs, compared to 82% of CG GPs, viewed the treatment suitable for bridging the waiting period until psychotherapy.

All IG GPs stated that the treatment met patients’ needs, was applicable in daily life, supported emotional coping and enhanced well-being. Except for emotional coping, in the CG, agreement was generally lower – met patients’ needs: 82%, applicable in daily life: 91%, enhanced well-being: 91%.

##### Acceptability-related patient responses:

In both groups, all patients indicated that they were motivated for the treatment. Apart from one IG patient, all patients (IG: 98%, CG: 100%) reported being satisfied with their GP’s psychological support. Agreement with the treatment content was high in both groups (IG: 96%, CG: 100%), as was agreement with the helpfulness of each of the four sessions (IG: 87–94%, CG: 84–90%). All IG patients expressed satisfaction with the delivery format (vs. CG: 93%), considered the treatment structure logical (vs. CG: 77%) and the content understandable (vs. CG: 87%).

Among IG patients, all but one patient (98%) found the flipchart helpful. Most regarded the handbook materials as helpful (93%) and satisfactory (95%); all agreed that they were easy to understand. Only 45% endorsed the idea of supplementing the treatment with digital tools in the future.

Most patients in both groups (IG: 91%, CG: 94%) felt that the treatment was helpful for improving emotional coping, while a higher share of CG patients (94% vs. IG: 85%) agreed that it enhanced well-being.

#### Treatment maintenance

##### GPs’ perspective:

The treatment’s long-term potential to enhance psychological services in PC was more frequently recognised by IG GPs (92% vs. CG: 82%). A greater proportion of IG GPs also stated they would recommend the treatment to colleagues (93% vs. CG: 82%), while willingness to offer the treatment again was more often reported by CG GPs (91% vs. IG: 84%).

##### Patients’ perspective:

More IG patients (91% vs. CG: 81%) viewed the treatment as helpful for managing psychological difficulties in the long term. In both groups, most patients agreed that they would recommend the treatment (94% each) and would be willing to repeat it (IG: 91%, CG: 93%).

#### Treatment-related unintended consequences

All GPs stated that the treatment caused no negative side effects in patients. Accordingly, most patients in both groups agreed (IG: 91%, CG: 90%).

#### Overalltreatment satisfaction

IG GPs gave the treatment a more favourable average rating than CG GPs (M = 1.62, *SD* = 0.96 vs. CG: M = 2.64, *SD* = 0.50). Among patients, both groups were similarly satisfied (IG: M = 1.62, *SD* = 0.89; CG: M = 1.57, *SD* = 0.86).

### Potential effectiveness

#### Transdiagnostic outcomes

**Fig. 5:**
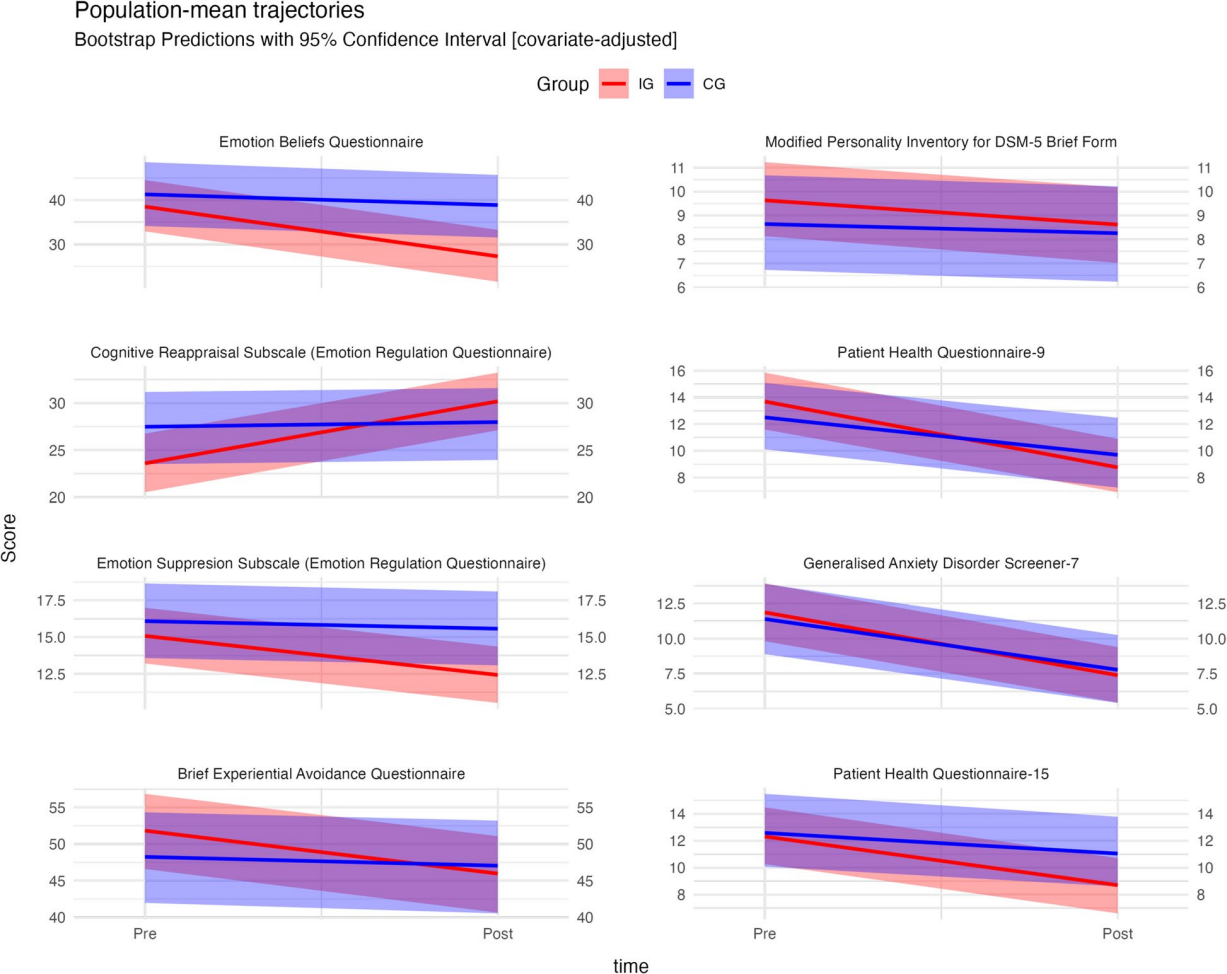
Pre-post changes in transdiagnostic and diagnosis-specific outcomes over time. Legend: IG = Intervention group, CG = Control group; adjusted covariates: age, gender and level of education

The most pronounced group differences over time – in favour of the IG – were found for a reduction in maladaptive beliefs (EBQ: −8.89, 95% CI [−15.10, −2.74]), an increase in cognitive reappraisal (ERQ subscale: 6.03, 95% CI [2.52, 9.55]) and a reduction in experiential avoidance (BEAQ: −4.57, 95% CI [−8.69, 0.09]). Notably, the CI of the latter included zero (Fig. 5). The corresponding ICCs were 0.14 (95% CI [0.00, 0.35]), 0.00 (95% CI [0.00, 0.13]) and 0.12 (95% CI [0.00, 0.33]), respectively; the ICC for cognitive reappraisal indicated no detectable clustering at the practice level.

Further group differences over time were observed for reductions in emotion suppression (ERQ subscale: −2.11, 95% CI [−4.41, 0.40]) and negative affectivity (PID5BF + M subscale: −0.58, 95% CI [−1.74, 0.57]). Of note, the CIs of both outcomes included zero (Fig. 5). The associated ICCs were 0.04 (95% CI [0.00, 0.21]) and 0.04 (95% CI [0.00, 0.23]), respectively.

#### Diagnosis-specific outcomes

For diagnosis-specific outcomes, the estimated group differences over time were: −2.08 for depressive symptoms (PHQ-9: 95% CI [−4.49, 0.06]; ICC: 0.00, 95% CI [0.00, 0.12]), −0.83 for anxiety symptoms (GAD-7: 95% CI [−2,85, 1.20]; ICC: 0.00, 95% CI [0.00, 0.13]) and −2.13 for somatoform symptoms (PHQ-15: 95% CI [−4.09, 0.11]; ICC: 0.00, 95% CI [0.00, 0.00]), with the CIs of all three outcomes including zero (Fig. 5). The corresponding ICCs were estimated as zero, indicating no observable variance at the practice level.

Among the 67 patients (IG: *n* = 40; CG: *n* = 27)^3^ who met baseline caseness criteria on at least one of the PHQ-9, GAD-7 or PHQ-15 measures, recovery was observed in 50% of IG patients (20/40) compared to 37% of CG patients (10/27). Reliable improvement was achieved by 67.5% of IG patients (27/40) versus 51.9% of CG patients (14/27), while reliable recovery was found in 40% of IG patients (16/40) compared to 22.2% of CG patients (6/27). Reliable deterioration occurred infrequently in both groups, affecting 2.5% of IG patients (1/40) and 7.4% of CG patients (2/27).

Additional file 2 contains within-group changes in transdiagnostic and disorder-specific outcomes over time, as well as PACIC outcomes and mean PC-PTSD-5 scores.

## Discussion

PC plays a central role in managing mental health conditions [8, 43], yet GPs are often faced with structural challenges inherent to this setting [43] and the complexity of patients presenting with non-specific, comorbid symptoms [11, 15]. A transdiagnostic approach may help address these issues and has shown positive outcomes in PC settings [21]. However, research on the delivery of interventions by GPs remains scarce. Therefore, this pilot study evaluated a GP-led, UP-based transdiagnostic intervention in terms of feasibility, acceptability and potential effectiveness.

### Feasibility and acceptability

Patient drop-out was lower (IG: 12%, CG: 11%) than in other PC-based brief psychological intervention studies [78]. Meanwhile, although the recruitment target of 20 practices was met, the intended sample of 100 patients was not reached, potentially affecting the precision of estimated rates [69]. Notably, no consensus exists on sample size requirements for pilot studies [79], and the achieved patient number aligns with other clinical pilot studies reporting median sample sizes of 30–36 per arm [80].

Although multi-session formats may pose an implementation barrier in PC [81], most GPs considered the treatment suitable for PC (IG: 92%, CG: 91%). However, more IG GPs than CG GPs felt it could be easily integrated into daily care (93% vs. CG: 63%) and serve as a bridge to psychotherapy (IG: 100%, CG: 82%). In addition, more IG GPs (69% vs. CG: 45%) perceived the overall time effort for treatment as adequate. These differences may reflect the challenges of applying clinical guidelines in PC. While the clinical guidelines that supplemented GPs’ usual care in the control group represent current best-practice standards for German medical care, clinical guidelines can be extensive and of limited relevance to comorbid symptom presentations due to their focus on a single condition [82]. Against this background, it is notable that a brief 2-h training was perceived as sufficient by IG GPs: nearly all (92%) felt adequately prepared for treatment delivery, despite this being shorter than training in comparable studies [25, 26, 83].

Interestingly, although many CG GPs considered the time effort too high, over a third (36%) still found four sessions too few – potentially reflecting the difficulties comorbidity creates for treatment planning [46]. Structured communication formats, such as the transdiagnostic intervention, have been linked to improved feasibility and acceptability [81]. Combined with its multiple-condition focus, this likely contributed to greater agreement among IG GPs with the treatment overall (85% vs. CG: 73%), its structure (100% vs. CG: 72%) and content (92% vs. CG: 72%).

Importantly, structured treatment formats require patients to understand and engage with the treatment rationale [84]. This may explain why, despite all IG patients evaluating the delivery format, treatment structure and content clarity positively – relative to 93%, 77% and 87% of CG patients, respectively – a slightly higher proportion of CG patients (94% vs. IG: 85%) agreed that the treatment improved well-being. Whereas iTAU likely permitted treatment flexibility, the more standardised transdiagnostic intervention may have offered limited opportunities for treatment personalisation [19] – a factor valued by patients [85] – thereby reducing acceptance among some.

Nevertheless, IG GPs unanimously agreed that the treatment addressed patients’ needs, was applicable to daily life, improved emotional coping and well-being, relative to lower levels of agreement among CG GPs (82—91%) – except for improved emotional coping, where both groups reported full agreement. Therefore, while both treatments were generally well received, the transdiagnostic intervention demonstrated an inclination for greater feasibility and acceptability than iTAU.

### Potential effectiveness

The observed trend towards greater improvement in transdiagnostic factors among IG patients compared to CG patients over time is consistent with findings from another PC-based study, in which disorder-specific treatment failed to significantly improve emotion regulation difficulties in two of three patient groups [86].

Notably, the three core modules of the intervention – beliefs about emotions, cognitive reappraisal and experiential avoidance – showed the largest observed between-group differences over time (the CI of experiential avoidance included zero). This adds to the literature on modularised UP approaches, suggesting that targeting specific transdiagnostic factors can yield improvements in corresponding outcomes [42, 87].

Addressing beliefs about emotions is considered an important foundation for developing emotion regulation skills [36, 42], while cognitive flexibility and experiential avoidance have been identified as potential mediators of positive treatment outcome [88]. Still, the overlap among transdiagnostic factors complicates isolating the unique impact of one mechanism [88–90]. Treatment-related improvements are therefore likely to reflect the combined effects of multiple, interacting emotion regulation strategies [90].

Following recent calls to investigate links between higher-order psychopathology (e.g., negative affectivity) and lower-order mental health symptoms [91, 92], these preliminary findings imply that focusing treatment on transdiagnostic factors may help improve patients’ symptomatology [88, 93] – beyond effects attributable to therapeutic alliance [85]. This is further supported by 67.5% (27/40) of IG patients achieving reliable improvement and 40% (16/40) reaching reliable recovery, rates that appear comparable to those reported for similar interventions in PC settings [5, 22]. Of note, previous UP-based interventions have already shown significant reductions in symptom severity in PC patients [47, 48]. Collectively, this supports the case for re-evaluation of the transdiagnostic intervention in a fully powered main trial.

### Implications for further research

Overall, the high acceptability and feasibility of the transdiagnostic intervention suggest particular promise for its future implementation as psychological first aid in PC. The findings indicate that minimal training is sufficient for GPs to effectively deliver a structured transdiagnostic treatment supplemented by patient homework materials. Together, these components may create a low-threshold approach that could support more consistent treatment delivery and patient engagement while reducing implementation barriers in routine care. By offering early GP-delivered psychological support, this approach could play a pivotal role within stepped-care models [94], particularly in addressing delays in access to specialist psychotherapy. Given these promising implications, three key considerations should guide future research to further evaluate and optimise this approach:

First, there is a need to improve recruitment strategies, as only 9% of contacted practices participated – lower than in comparable studies (16–44%; [25, 27, 83]) – and patient recruitment fell short of target. At practice level, strategies such as offering CME credits [95] and using phased, marketing-inspired campaigns [96] may enhance engagement. For patient recruitment, public awareness strategies, such as social media outreach [97], waiting room posters/flyers [98] or personalised invitations informed by practices’ patient record systems [25] may encourage more patients to approach their GP. However, operational and financial constraints [4], such as the volume-based reimbursement structure [8], will likely remain a recruitment barrier. In addition, stigma-related hesitation [85] may have contributed to roughly one-quarter of patients declining participation.

Second, the transdiagnostic intervention should be streamlined to better fit the default 20-min session length, which 54% of IG GPs considered too short for content delivery. This may explain why a higher proportion of CG GPs (91% vs. IG: 84%) said they would offer the treatment again, despite IG GPs rating its potential for PC (92% vs. CG: 82%) and the likelihood of recommending it to colleagues (93% vs. CG: 82%) higher. In this context, a blended care format could improve the intervention’s prospects [19] and further reduce GP workload [99], while also meeting some patients’ desire for more (IG: 30%, CG: 42%) and longer sessions (IG: 22%, CG: 26%). Given that 23% of IG GPs expressed dissatisfaction with the final session, digital components could for example provide supplementary relapse prevention [100, 101], thereby enhancing treatment closure. However, scepticism about digital tools among nearly a third of IG patients (29%) suggests that well-received analogue elements – such as the flipchart and handbook – should be retained.

Third, outcome assessment should be expanded. Including a follow-up assessment would help distinguish intervention effects from spontaneous remission [102] and provide insight into the sustainability of treatment effects. Session-by-session outcome monitoring could additionally support GPs by offering timely feedback [14] and help reduce drop-out-related bias in data analysis [5]. Also, for a more granular understanding of the dynamics in transdiagnostic and disorder-specific outcome changes, ecological momentary assessment may offer added value [103, 104]. Finally, systematic assessment of concurrent pharmacological treatment, including medication type, dosage, duration and adherence, should be incorporated to account for potential confounding of treatment effects.

Next to a fully powered main trial, future research could further investigate transdiagnostic mechanisms of change by examining whether a specific component of the transdiagnostic treatment plays a particularly important role in symptom improvement. Comparable work has already been conducted in the context of an internet-delivered transdiagnostic intervention [88]. Moreover, it may be valuable to evaluate the screening potential of the utilised transdiagnostic mechanisms, that is, to determine which mechanism best captures overall symptom burden and may therefore be most informative for treatment conceptualisation [39, 105]. Building on the concept of a modularised UP delivery [42], such findings could help inform more personalised interventions tailored to patients’ specific strengths and deficits in emotion regulation [106]. However, the complexity of personalised treatment may exceed the time-limited resources of routine PC [43]. Accordingly, future studies should also examine implementation models that broaden treatment delivery beyond exclusively GP-led sessions. In addition to digital augmentation, this could include task-sharing approaches [107], such as redistributing selected therapeutic tasks to patients' significant others. Finally, health-economic evaluations are needed to determine whether transdiagnostic treatment approaches offer financial or resource-related advantages over usual care.

### Strengths and limitations

This pilot study addressed a key research gap by comprehensively evaluating the feasibility, acceptability and potential effectiveness of a GP-led, transdiagnostic intervention. Following an established process-evaluation framework [58], it assessed both diagnosis-specific and transdiagnostic outcomes. Further strengths include the adaptation of the intervention from the evidence-based UP [31], the alignment of iTAU with official clinical treatment guidelines to ensure standardisation while maintaining external validity [108] and the controlling for therapeutic alliance effects [10] using identical treatment durations for both groups. Also, stratified cluster randomisation at practice level prevented contamination and ensured balanced GP allocation between groups [109, 110].

Several limitations warrant consideration: Generalisability of results is restricted by the regional focus (Munich, Germany) and the predominance of research-experienced practices familiar with applying novel treatment approaches. Due to the lack of follow-up data, symptom changes may reflect spontaneous remission [111] with the sustainability of effects remaining unclear. Furthermore, the impossibility of participant blinding may have introduced bias. In addition, differences in GPs’ professional experience between groups were not statistically adjusted. Whilst therapist experience has not been shown to be consistently related to treatment outcome [112], a potential influence on the quality of treatment delivery cannot be excluded. Besides, thresholds for reliable improvement and reliable deterioration were adopted from a comparable study [22] rather than calculated from study-specific parameters due to the limited power of this pilot study. Although the referenced study involved similar patient and study characteristics, this approach may have reduced indices precision. Also, monitoring of concurrent medication prescriptions was partly incomplete and based on patients’ self-reports, precluding robust analytical adjustment for psychopharmacological confounding. Finally, the general reliance on self-report measures [113], the small sample size and unequal cluster sizes [69] may limit conclusions about treatment estimates.

## Conclusion

This pilot study supports the feasibility and acceptability of a GP-led, UP-based transdiagnostic intervention in PC. Observed improvements in transdiagnostic factors indicate its potential to promote positive symptom change. Overall, this underscores the intervention’s utility as psychological first aid and supports the case for a fully powered main trial. In preparation, it appears recommendable to refine recruitment strategies, streamline the intervention for flexible delivery and implement more comprehensive outcome tracking. Advancing transdiagnostic approaches in PC may offer scalable, low-threshold options to address the mental health treatment gap.

## Supplementary Information

Additional file 1: Treatment feasibility and acceptability questionnaires completed by general practitioners and patients.

Additional file 2: Within group changes in transdiagnostic and diagnosis-specific outcomes over time, as well as PACIC outcomes and PC-PTSD-5 mean scores.

## Data Availability

The datasets generated and analysed during the current study, together with the statistical code, are available in the Open Science Framework (OSF) repository: 10.17605/OSF.IO/96U34.

## Abbreviations

BEAQ: Brief Experiential Avoidance Questionnaire
CG: Control Group
CI: Confidence Interval
CONSORT: Consolidated Standards of Reporting Trials
DRKS: Deutsches Register Klinischer Studien
EBQ: Emotion Beliefs Questionnaire
ERQ: Emotion Regulation Questionnaire
GAD-7: Generalised Anxiety Disorder Screener-7
GP: General Practitioner
IG: Intervention Group
iTAU: Improved Treatment as Usual
K-6: Kessler Psychological Distress Scale-6
LMM: Linear Mixed Model
PACIC: Patient Assessment of Chronic Illness Care
PC: Primary Care
PHQ-15: Patient Health Questionnaire-15
PHQ-9: Patient Health Questionnaire-9
PID5BF + M: Modified Personality Inventory for DSM-5 Brief Form
REML: Restricted Maximum Likelihood Estimation
SPIRIT: Standard Protocol Items: Recommendations for Interventional Trials
UP: Unified Protocol

## Footnotes

1 Psychosomatic care is a required training for German GPs in basic mental health care.

2 In this and future instances, “agree” and similar expressions (e.g., “reported”, “indicated”) refer to the combined percentage of participants who selected “rather agree” or “strongly agree”.

3 Only 67 of the 77 treatment completers met PHQ-9, GAD-7 and/or PHQ-15 caseness as initial study inclusion was based on Kessler-6 screening and GP clinical judgement rather than diagnosis specific thresholds.
