# Supplementary material for: Evaluation of a transdiagnostic mental health intervention in German primary care: a parallel-group, two-arm, cluster randomised controlled pilot study
Source: BMC Prim Care. 2026 May 21;27:228. doi: 10.1186/s12875-026-03377-4 (PMC13251099; doi:10.1186/s12875-026-03377-4)
Supplement: Supplementary file 1 — Additional file 1: Treatment feasibility and acceptability questionnaires completed by general practitioners and patients. [file 12875_2026_3377_MOESM1_ESM.docx]

# Additional file 1. Treatment feasibility and acceptability questionnaires completed by general practitioners and patients

**Feasibility and acceptability questionnaire completed by GPs**

Tab. 1. General questions on feasibility and acceptability

|  | **Strongly disagree** | **Rather disagree** | **Rather agree** | **Strongly agree** |
| --- | --- | --- | --- | --- |
| The treatment was suitable for use in primary care. |  |  |  |  |
| *The training sufficiently prepared me to implement the treatment.* |  |  |  |  |
| My psychological knowledge was sufficient to apply the treatment. |  |  |  |  |
| The treatment differed from my usual approach to treating mental health conditions.  Additional open-ended question   - Rather/strongly agree:  In what ways did the treatment differ from your usual approach to mental health care? |  |  |  |  |
| The treatment could be easily integrated into daily practice. |  |  |  |  |
| Overall, I was satisfied with the treatment. |  |  |  |  |
| The treatment followed a logical structure. |  |  |  |  |
| I was satisfied with the treatment content. |  |  |  |  |
| *The flipchart was helpful for delivering the treatment.* |  |  |  |  |
| *I was satisfied with the first treatment session.* |  |  |  |  |
| *I was satisfied with the second treatment session.* |  |  |  |  |
| *I was satisfied with the third treatment session.* |  |  |  |  |
| *I was satisfied with the fourth treatment session.* |  |  |  |  |
| Patient willingness to participate in the treatment was high.  Additional open-ended questions   - Rather/strongly agree: Please describe which recruitment methods were particularly effective. - Rather/strongly disagree: Which recruitment methods could enhance recruitment success? |  |  |  |  |
| Recruited patients were representative of patients who usually present in primary care with mental health conditions.  Additional open-ended question   - Rather/strongly disagree: Please explain why recruited patients were (rather) not representative. |  |  |  |  |
| I adhered to the guidelines for implementing the treatment.  Additional open-ended questions   - Rather/strongly agree:  Please indicate whether you have implemented any further treatment measures. - Rather/strongly disagree: Please describe what alternative treatment measures you implemented. |  |  |  |  |
| The treatment was suitable as psychological first aid. |  |  |  |  |
| The treatment was suitable to bridge the waiting time for psychotherapy (if applicable). |  |  |  |  |
| The treatment encouraged patients to complete the full treatment duration. |  |  |  |  |
| The treatment content was applicable to patients’ everyday life. |  |  |  |  |
| The treatment addressed patients’ needs. |  |  |  |  |
| The treatment helped patients to better cope with their mental health condition. |  |  |  |  |
| The treatment approach had a positive effect on patients’ well-being. |  |  |  |  |
| I would be willing to administer the treatment again. |  |  |  |  |
| I would recommend the treatment to colleagues. |  |  |  |  |
| The treatment has a long-term potential to improve psychological services in primary care. |  |  |  |  |
| The treatment did not lead to any negative side effects in patients. |  |  |  |  |

*Note.* *Italic* = questions only answered by general practitioners of the intervention group.

Tab. 2. Questions on time investment

|  | **Too short / low** | **Adequate / sufficient** | **Too long / high** |
| --- | --- | --- | --- |
| The overall time effort for the treatment (excluding study-related tasks) was... |  |  |  |
| The default of 20 minutes per treatment session was... |  |  |  |
| The number of 4 treatment sessions was... |  |  |  |

Tab. 3. Question on overall treatment satisfaction

|  | **1 (Very good)** | **2** | **3** | **4** | **5** | **6 (Very bad)** |
| --- | --- | --- | --- | --- | --- | --- |
| How would you rate the treatment overall? |  |  |  |  |  |  |

**Feasibility and acceptability questionnaire completed by patients**

Tab. 4. General questions on feasibility and acceptability

|  | **Strongly disagree** | **Rather disagree** | **Rather agree** | **Strongly agree** |
| --- | --- | --- | --- | --- |
| My motivation to participate in the treatment was high. |  |  |  |  |
| I was satisfied with the psychological support provided by my GP during the treatment. |  |  |  |  |
| I was satisfied with how the treatment was delivered. |  |  |  |  |
| The treatment followed a logical structure. |  |  |  |  |
| I was satisfied with the content of the treatment sessions. |  |  |  |  |
| The content of the treatment sessions was understandable to me. |  |  |  |  |
| *The flipchart was helpful in understanding the session content.* |  |  |  |  |
| The first treatment session was helpful to me. |  |  |  |  |
| The second treatment session was helpful to me. |  |  |  |  |
| The third treatment session was helpful to me. |  |  |  |  |
| The fourth treatment session was helpful to me. |  |  |  |  |
| *I was satisfied with the handbook's texts and exercises.* |  |  |  |  |
| *The handbook's texts and exercises were understandable to me.* |  |  |  |  |
| *The handbook's texts and exercises were helpful to me.* |  |  |  |  |
| *The time required to read the texts and complete the exercises in the handbook was acceptable to me.* |  |  |  |  |
| The treatment helped me cope better with my mental health condition. |  |  |  |  |
| The treatment had a positive effect on my well-being. |  |  |  |  |
| I am confident that, in the long term, the treatment will help me cope better with my mental health condition. |  |  |  |  |
| I would recommend the treatment (e.g., to friends). |  |  |  |  |
| I would choose to receive the treatment again. |  |  |  |  |
| The treatment did not lead to any negative side effects. |  |  |  |  |
| *I would find it helpful if the treatment was supplemented by digital components (e.g., an app, website).* |  |  |  |  |

*Note.* *Italic* = questions only answered by patients of the intervention group.

Tab. 5. Questions on time investment

|  | **Too short / few** | **Adequate / sufficient** | **Too long / too many** |
| --- | --- | --- | --- |
| I found the length of the individual treatment sessions to be... |  |  |  |
| I found the number of treatment sessions to be... |  |  |  |

Tab. 6. Question on overall treatment satisfaction

|  | **1 (Very good)** | **2** | **3** | **4** | **5** | **6 (Very bad)** |
| --- | --- | --- | --- | --- | --- | --- |
| How would you rate the treatment overall? |  |  |  |  |  |  |
