# Supplementary material for: Evaluation of a transdiagnostic mental health intervention in German primary care: a parallel-group, two-arm, cluster randomised controlled pilot study
Source: BMC Prim Care. 2026 May 21;27:228. doi: 10.1186/s12875-026-03377-4 (PMC13251099; doi:10.1186/s12875-026-03377-4)
Supplement: Supplementary file 2 — Additional file 2: Within group changes in transdiagnostic and diagnosis-specific outcomes over time, as well as PACIC outcomes and PC-PTSD-5 mean scores. [file 12875_2026_3377_MOESM2_ESM.docx]

**Additional file 2.** Within group changes in transdiagnostic and diagnosis-specific outcomes over time, as well as PACIC outcomes and PC-PTSD-5 mean scores

Tab. 1. Within group changes in transdiagnostic and diagnosis-specific outcomes over time (covariate adjusted)

| Variable | Group | Median | CI low (2.5%) | CI high (97.5%) |
| --- | --- | --- | --- | --- |
| Transdiagnostic outcomes | | | | |
| Emotion Beliefs Questionnaire | | | | |
|  | IG | -11.23 | -15.38 | -7.46 |
|  | CG | -2.33 | -7.48 | 2.55 |
| Emotion Regulation Questionnaire | | | | |
| Cognitive reappraisal subscale | IG | 6.55 | 4.30 | 8.91 |
|  | CG | 0.62 | -2.25 | 3.44 |
| Emotion suppression subscale | IG | -2.60 | -4.15 | -1.17 |
|  | CG | -0.51 | -2.36 | 1.41 |
| Brief Experiential Avoidance Questionnaire | | | | |
|  | IG | -5.94 | -8.54 | -3.13 |
|  | CG | -1.34 | -4.73 | 1.97 |
| Modified Personality Inventory for  DSM-5 Brief Form | | | | |
| Negative affectivity subscale | IG | -1.00 | -1.69 | -0.22 |
|  | CG | -0.38 | -1.27 | 0.51 |
| Diagnosis-specific outcomes | | | | |
| Patient Health Questionnaire-9 | | | | |
|  | IG | -4.92 | -6.31 | -3.49 |
|  | CG | -2.75 | -4.65 | -1.06 |
| Generalised Anxiety Disorder Screener-7 | | | | |
|  | IG | -4.47 | -5.80 | -3.20 |
|  | CG | -3.65 | -5.24 | -1.98 |
| Patient Health Questionnaire-15 | | | | |
|  | IG | -3.57 | -5.03 | -2.44 |
|  | CG | -1.51 | -3.10 | 0.06 |

*Note.* *n* = sample size, IG = Intervention group, CG = Control group, CI = Confidence interval; adjusted covariates: age, gender and level of education.

Tab. 2. PACIC outcomes for the intervention group and control group

| Subscale/Item | Group | Count | | | |
| --- | --- | --- | --- | --- | --- |
|  | IG (*n* = 16) CG (*n* = 20) | 0 –  25% | 26 – 50% | 51 – 75% | 76 – 100% |
| 1) Patient Activation |  |  |  |  |  |
| Treatment options | IG | 4 | 4 | 3 | 5 |
|  | CG | 7 | 5 | 5 | 3 |
| 2) Delivery System Design and decision support |  |  |  |  |  |
| Organisation of care | IG | 2 | 2 | 5 | 7 |
|  | CG | 5 | 3 | 6 | 6 |
| 3) Goal setting and tailoring |  |  |  |  |  |
| Goal setting | IG | 3 | 3 | 2 | 8 |
|  | CG | 6 | 5 | 3 | 6 |
| Written treatment plan | IG | 7 | 2 | 1 | 6 |
|  | CG | 9 | 3 | 1 | 7 |
| Group referral | IG | 1 | 3 | 2 | 10 |
|  | CG | 7 | 4 | 4 | 5 |
| Health-related behaviour | IG | 8 | 2 | 1 | 5 |
|  | CG | 10 | 3 | 3 | 4 |
| 4) Problem-solving and contextual counselling |  |  |  |  |  |
| Feasible treatment planning | IG | 7 | 2 | 1 | 6 |
|  | CG | 9 | 6 | 3 | 2 |
| Preparation for difficult times | IG | 7 | 2 | 0 | 7 |
|  | CG | 9 | 2 | 4 | 5 |
| Impact on life | IG | 4 | 2 | 3 | 7 |
|  | CG | 9 | 2 | 2 | 7 |
| 5) Follow-up and coordination |  |  |  |  |  |
| Practice follow-up | IG | 3 | 1 | 4 | 8 |
|  | CG | 8 | 5 | 1 | 6 |
| Care coordination | IG | 9 | 0 | 2 | 5 |
|  | CG | 10 | 2 | 4 | 4 |

*Note.* *n* = sample size, IG = Intervention group, CG = Control group.

Tab. 3. Mean PC-PTSD-5 scores of the intervention group and control group

| Measure | Group  IG (*n* = 32) CG (*n* = 22) | Mean (*SD*) |
| --- | --- | --- |
| Primary Care Post-Traumatic Stress Disorder-5 | IG | 1.94 (*1.81*) |
|  | CG | 2.09 (*1.63*) |

*Note.* *n* = sample size, IG = Intervention group, CG = Control group, *SD* = Standard Deviation; Due to an error in questionnaire design, the PC-PTSD-5 was assessed from patients at t_1_ instead of t_0_, as was specified in the study protocol.
